# Solitary wave solutions of the fourth order Boussinesq equation through the exp(–Ф(*η*))-expansion method

**DOI:** 10.1186/2193-1801-3-344

**Published:** 2014-07-08

**Authors:** M Ali Akbar, Norhashidah Hj Mohd Ali

**Affiliations:** Department of Applied Mathematics, University of Rajshahi, Rajshahi, Bangladesh; School of Mathematical Sciences, Universiti Sains Malaysia, Pulau Pinang, Malaysia

**Keywords:** exp(–Ф(*η*))-expansion method, Fourth order Boussinesq equation, Solitary wave solutions, Soliton, Traveling wave solutions

## Abstract

**Abstract:**

The exp(–Ф(*η*))-expansion method is an ascending method for obtaining exact and solitary wave solutions for nonlinear evolution equations. In this article, we implement the exp(–Ф(*η*))-expansion method to build solitary wave solutions to the fourth order Boussinesq equation. The procedure is simple, direct and useful with the help of computer algebra. By using this method, we obtain solitary wave solutions in terms of the hyperbolic functions, the trigonometric functions and elementary functions. The results show that the exp(–Ф(*η*))-expansion method is straightforward and effective mathematical tool for the treatment of nonlinear evolution equations in mathematical physics and engineering.

**Mathematics subject classifications:**

35C07; 35C08; 35P99

## Background

The world around us is inherently nonlinear (He 2009) and nonlinear evolution equations (NLEEs) are widely used as models to describe complex physical phenomena in various fields of science and engineering, especially in solid-state physics, plasma physics, fluid mechanics, biology etc. One of the fundamental problems for these models is to obtain their travelling wave solutions as well as solitary wave solutions. In particular, various methods have been utilized to explore different kinds of solutions of physical problems described by nonlinear evolution equations. In the numerical methods, stability and convergence should be considered, so as to avoid divergence or inappropriate results. However, in recent times, a variety of analytical and semi-analytical methods have been developed and use for solving NLEEs, for instance, the inverse scattering transform (Ablowitz and Clarkson 1991), the complex hyperbolic function method (Chow 1995; Zayed et al. 2006), the rank analysis method (Feng 2000), the ansatz method (Hu 2001a, [b]), the (*G*′/*G*)-expansion method (Wang et al. 2008; Bekir 2008; Neyrame et al. 2012; Akbar et al. 2012; Alam and Akbar 2013; Alam et al. 2014), the Exp-functions method (He and Wu 2006), the modified simple equation method (Jawad et al. 2010; Khan et al. 2013), the Jacobi elliptic function method (Chen and Wang 2005; Liu 2005), the Adomian decomposition method (Adomian 1994; Wazwaz 2002), the homogeneous balance method (Wang 1995; Zayed et al. 2004), the F-expansion method (Wang and Zhou 2003; Wang and Li 2005), the Backlund transformation method (Miura 1978), the Darboux transformation method (Matveev and Salle 1991), the homotopy perturbation method (Mohyud-Din 2007; Mohyud-Din and Noor 2009), the generalized Riccati equation method (Yan and Zhang 2001), the tanh-function method (Wazwaz 2005), the Hirota’s bilinear method (Hirota 2004), the auxiliary equation method (Sirendaoreji 2007), the exp(–Ф(*η*))-expansion method (Khan and Akbar 2013) etc.

The objective of this article is to implement the potential exp(–Ф(*η*))-expansion method to search solitary wave solutions for nonlinear evolution equations via the fourth order Boussinesq equation. In former literature, the solitary wave solutions to the Boussinesq equation have not been studied by this method.

The article is organized as follows: In Methodology Section, we give the description of the exp(–Ф(*η*))-expansion method. We apply this method to the fourth order Boussinesq equation in the Application Section. 2D and 3D graphs are given in the Graphical Representations of the Solutions Section. Finally, in Conclusion Section, we draw our conclusions.

## Methodology

Let us consider the nonlinear evolution equation in the form
1

where *u* = *u*(*x*, *t*) is an unknown function, *F* is a polynomial in *u*(*x*, *t*) and its derivatives in which highest order derivatives and nonlinear terms are involved and the subscripts indicate partial derivatives. In order to investigate solitary wave solutions of (1) by using the exp(–Ф(*η*))-expansion method, we have to perform the following important steps:

Step 1. We combine the real variables *x* and *t* by a compound variable *η*2

where *V* is the celerity of the traveling wave. By means of traveling wave transformation (2), Eq. (1) switch into an ordinary differential equation (ODE) for *u* = *u*(*η*):
3

where *H* is a polynomial of *u* and its derivatives and the superscripts refer to the ordinary derivatives with respect to *η*.

Step 2. Assume the traveling wave solution of (3) can be articulated as follows:
4

*A*_*i*_ (0 ≤ *i* ≤ *N*) are constants to be determined, such that *A*_*N*_ ≠ 0 and Ф = Ф(*η*) satisfies the following auxiliary equation:
5

Depending on the parameters involved, Eq. (5) has the subsequent solutions:

When *μ* ≠ 0, and *λ*^2^ - 4*μ* > 0, 
6

When *μ* ≠ 0, and *λ*^2^ - 4*μ* < 0, 
7

When *μ* = 0, *λ* ≠ 0, and *λ*^2^ - 4*μ* > 0, 
8

When *μ* ≠ 0, *λ* ≠ 0, and *λ*^2^ - 4*μ* = 0, 
9

When *μ* = 0, *λ* = 0, and *λ*^2^ - 4*μ* = 0, 
10

Step 3. The positive integer *N* can be determined by considering the balance between the highest order derivatives and the nonlinear terms of the highest order appearing in (3).

Step 4. We substitute Eq. (4) into Eq. (3) and then we take into consideration the function exp(–Ф(*η*)). In consequence of this substitution, we obtain a polynomial in exp(–Ф(*η*)). We collect all the coefficients of identical power of exp(–Ф(*η*)) and equalize to zero delivers a system of algebraic equations whichever can be solved to find *A*_*N*_, ⋯⋯, *V*, *λ*, *μ*. The values of *A*_*N*_, ⋯⋯, *V*, *λ*, *μ* along with general solutions of Eq. (5) complete the determination of the solution of Eq. (1).

## Application

In this section, we will use the exp(–Ф(*η*))-expansion method to construct the exact solutions and then the solitary wave solutions to the fourth order Boussinesq equation. Let us consider the equation
11

The above model (11) was introduced by Boussinesq to illustrate the propagation of long waves in shallow water (Lai et al. 2008), where *u*(*x*, *t*) is the elevation of the free surface of the fluid, where the subscripts denoting partial derivatives. The equation also arises in many other physical applications, such as, nonlinear lattice waves, iron sound waves in plasma, and vibrations in a nonlinear string. It was also applied to the study of the percolation of water in porous subsurface strata.

Equation (11) possesses solitary waves, extract from traveling wave solutions and Boussinesq was the first who gave a scientific explanation of their existence. We utilize the traveling wave variable *u*(*η*) = *u*(*x*, *t*), *η* = *x* - *Vt* and this operation changes (11) to the following ODE:
12

Integrating Eq. (12) twice with respect to *η* yields:
13

where *C* is an integration constant to be determined.

Balancing the highest order nonlinear term *u*^2^ and linear term of the highest order *u*^′′^ appearing in (13), yields *N* = 2. Therefore, the solution of Eq. (13) takes the form
14

where *A*_0_, *A*_1_, *A*_2_ are arbitrary constants such that *A*_2_ ≠ 0.

We substitute Eq. (14) into Eq. (13) and taking consideration Eq. (5), it generates a polynomial and then setting the coefficients of exp(–Ф(*η*)) to zero, yields
17181920

Solutions of Eqs. (17)-(20), yield


where *λ*, *μ* and *A*_0_ are arbitrary constants.

Substituting the values of *V*, *A*_0_, *A*_1_, *A*_2_ into Eq. (14), yields
21

where 

By using the solutions of Eq. (5) into Eq. (21), we obtain the succeeding traveling wave solutions of the fourth order Boussinesq equation:

Type 1: When *μ* ≠ 0, *λ*^2^ - 4*μ* > 0, 


where  and *E* is an arbitrary constant.

Type 2: When *μ* ≠ 0, *λ*^2^ - 4*μ* < 0, 


where  and *E* is an arbitrary constant.

Type 3: When *μ* = 0, *λ* ≠ 0, and *λ*^2^ - 4*μ* > 0, 


where  and *E* is an arbitrary constant.

Type 4: When *μ* ≠ 0, *λ* ≠ 0, and *λ*^2^ - 4*μ* = 0, 


where  and *E* is an arbitrary constant.

Type 5: When *μ* = 0, *λ* = 0, and *λ*^2^ - 4*μ* = 0, 


where  and *E* is an arbitrary constant.

Solitary wave solutions represent an important type of solutions for nonlinear partial differential equations (PDEs) as many nonlinear partial differential equations have been found to have a variety of solitary wave solutions. It is familiar that searching of exact solutions of nonlinear partial differential equations plays a significant role in the study of nonlinear physical phenomena. Exact traveling wave solutions are useful for verifying the accuracy and stability of popular numerical schemes such as the finite difference and finite element methods. The solitary wave solutions obtained in this article are encouraging, applicable, and could be helpful in analyzing long wave propagation on the surface of a fluid layer under the action of gravity, iron sound waves in plasma, and vibrations in a nonlinear string.

## Graphical representation of the solutions

**Figure 1:**
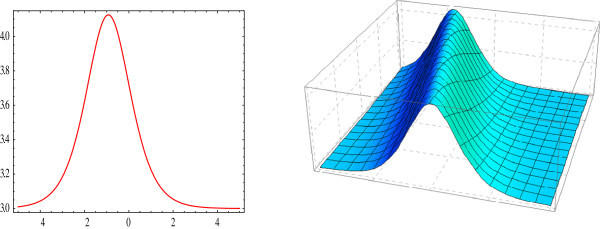
**Bell shaped 2D and 3D-plot of the solitary wave.** The graphs have been plotted from *u*_1_(*η*) when *μ* = 1, *λ* = 2.5, *E* = 0, *t* = 0 and *A*_0_ = 1.

**Figure 2:**
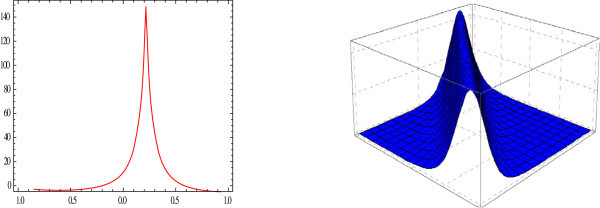
**Sharp bell shaped 2D and 3D-plot of the solitary wave.** The graphs have been plotted from *u*_2_(*η*) when *μ* = 2.5, *λ* = 1, *E* = 0, *t* = 0 and *A*_0_ = 1.

**Figure 3:**
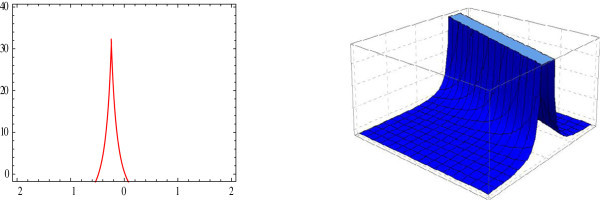
**Pointed 2D and 3D-plot of the solitary wave.** The graphs have been plotted from *u*_3_(*η*) when *μ* = 0, *λ* = 1, *E* = 1, *t* = 0 and *A*_0_ = 1.

**Figure 4:**
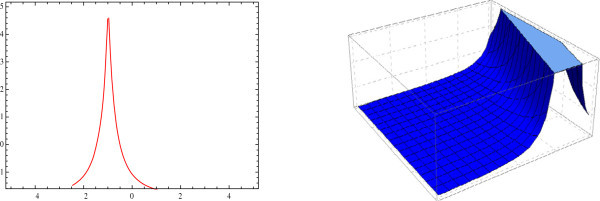
**Sharp bell shaped 2D and 3D-plot of the solitary wave.** The graphs have been plotted from *u*_4_(*η*) when *μ* = 0.25, *λ* = 1, *E* = 1, *t* = 0 and *A*_0_ = 1.

**Figure 5:**
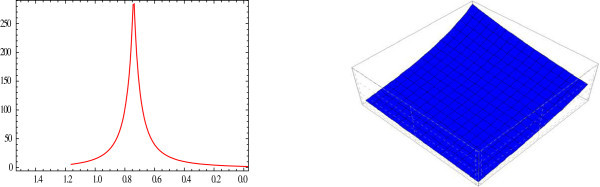
**2D and 3D-plot of the solitary wave.** The graphs have been plotted from *u*_5_(*η*) when *μ* = 0, *λ* = 0, *E* = 1, *t* = 0 and *A*_0_ = 1.

Solitary waves can be obtained from each traveling wave solution by setting particular values to its unknown parameters. By adjusting these parameters, one can get an internal localized mode. In this section, we have presented some graphs of solitary waves constructed by taking suitable values of the involved unknown parameters to visualize the underlying mechanism to the original physical phenomena. Using mathematical software Mathematica, two and three-dimensional plots of the obtained solutions have been shown in Figures 1, 2, 3, 4 and 5.

The obtained solutions of the fourth order Boussinesq equation incorporate four types of explicit solutions namely hyperbolic, trigonometric, exponential, and rational function solutions. From these explicit results we observe that solutions *u*_1_(*η*) and *u*_2_(*η*) are soliton and the rest of the three solutions are cuspon. The above solitary wave solutions might be useful in analyzing the propagation of long waves in shallow water, iron sound waves in plasma, and vibrations in a nonlinear string.

## Conclusions

In this article, we have successfully formulated solitary waves solutions from the traveling wave solutions to the fourth order Boussinesq equation through the exp(–Ф(*η*))-expansion method. The procedure is simple, direct and constructive with the help of a computer algebra system. The method is quite efficient and practically well suited to be used in finding solitary wave solutions of NLEEs and the attained solutions demonstrated the competence of the exp(–Ф(*η*))-expansion method. We also observed that the method is straightforward and can be applied to many other nonlinear evolution equations.
